# Modulation of peroxynitrite improves host response to vasopressin in ovine sepsis

**DOI:** 10.1186/cc12998

**Published:** 2013-11-05

**Authors:** Osamu Fujiwara, Yong Zhu, Daniel Traber, Lillian Traber, David Herndon, Perenlei Enkhbaatar

**Affiliations:** 1 Department of Anesthesiology, University of Texas Medical Branch, Galveston, TX, USA; 2 Shriners Hospital for Children, Galveston, TX, USA

## Background

The standard therapy for sepsis is becoming less effective due to increasing microorganism resistance to antibiotics and cardiovascular collapse refractory to fluid resuscitation and vasopressors. In this study, we demonstrate a critical role of peroxynitrite in vascular hyporesponsiveness to vasopressin (VP) in methicillin-resistant *Staphylococcus aureus *(MRSA)-induced ovine sepsis.

## Materials and methods

Sheep were instrumented with Swan Ganz (common jugular vein), femoral artery, and left atrium catheters to monitor hemodynamics for 24 hours. Sepsis was induced by instillation of live MRSA (2.5 to 3.5 × 10^11 ^CFU) into the lungs by bronchoscope under anesthesia. Sheep were then awakened, placed on a ventilator, and fluid resuscitated. Urine output was measured via a Foley catheter. Groups: MRSA, received MRSA, *n *= 4; MRSA + peroxynitrite decomposition catalyst (PDC), received MRSA and were treated with PDC starting 6 hours post injury (0.1 mg/kg bolus followed by 0.02 mg/kg/hour), *n *= 4; MRSA + VP, received MRSA and were titrated with VP when mean arterial pressure fell by 10 mmHg, *n *= 4; and MRSA + VP + PDC, received MRSA, treated with VP and PDC, *n *= 4.

## Results

MRSA induced severe hypotension refractory to aggressive fluid and AVP. PDC and AVP alone partially attenuated the severe hypotension. When combined they more effectively reversed the hypotension. Inhibition of peroxynitrite formation by PDC also markedly reduced AVP requirement. In addition, the *in vitro *effects of AVP (5 nM) on isolated arterial ring tone were abolished with co-incubation with peroxynitrite (50 μM).

## Conclusions

Peroxynitrite modulation may be a novel treatment option for management of sepsis-induced cardiovascular collapse refractory to vasopressors. These findings are especially provocative since peroxynitrite is the product of excessive nitric oxide regardless of which NOS isoform is involved and the major debate of whether the use of NOS inhibitors in management of sepsis is beneficial still remains.

